# A Comparison of Tablet Computer and Paper-Based Questionnaires in Healthy Aging Research

**DOI:** 10.2196/resprot.3291

**Published:** 2014-07-16

**Authors:** Jason Fanning, Edward McAuley

**Affiliations:** ^1^Exercise Psychology LaboratoryDepartment of Kinesiology and Community HealthUniversity of Illinois at Urbana-ChampaignUrbana, ILUnited States

**Keywords:** healthy aging, questionnaire, tablet computer, behavioral psychology

## Abstract

**Background:**

Digital questionnaire delivery offers many advantages to investigators and participants alike; however, evidence supporting digital questionnaire delivery via touchscreen device in the older adult population is lacking.

**Objective:**

The objective of this study was to compare the use of tablet computer-delivered and printed questionnaires as vehicles for the collection of psychosocial data from older adults to determine whether this digital platform would be readily adopted by the sample, and to identify whether tablet delivery influences the content of data received.

**Methods:**

The participants completed three questionnaires using both delivery methods, followed by a brief evaluation.

**Results:**

A nonparametric one-sample binomial test indicated a significantly greater proportion of individuals preferred the tablet-delivered questionnaires (*z*=4.96, SE 3.428, *P*<.001). Paired sample *t* tests and Wilcoxon sign-rank tests indicated that measures collected by each method were not significantly different (all *P*≥.273). Ease of use of the tablet interface and anxiety while completing the digital questionnaires were significantly correlated with preferences, (*r*_*s*_=.665, *P*<.001 and *r*_*s*_=.552, *P*<.001, respectively). Participants most frequently reported that the tablet delivery increased speed of use and improved data entry, although navigation was perceived as being more difficult. By comparison, participants felt that the paper packet was easier to read and navigate, but was slow and cumbersome, and they disliked the lack of dynamic features.

**Conclusions:**

This study provides preliminary evidence suggesting that questionnaires delivered to older adults using contemporary tablet computers may be acceptable and do not substantively influence the content of the collected data.

## Introduction

### Importance of Assessing Health-Related Quality of Life in Older Adults

The population of the United States is aging rapidly, and the number of adults 65 years of age or greater has increased more than 15% since the year 2000 [1]. Whereas life expectancy has increased, the onset of morbidity associated with advanced age has not been substantially delayed, resulting in many individuals living with disease for a great number of years [2]. Researchers are therefore targeting this population in an effort to increase health-related quality of life (HRQL) and reduce the financial burden associated with a larger portion of the population living with disease.

### Traditional Methods of Health-Related Quality of Life Assessment

The assessment of HRQL and health-related behavior is typically done by providing participants with paper and pencil questionnaires. For smaller sample sizes, this method is relatively inexpensive and can allow researchers to examine theoretical constructs that may underlie behavior change brought about by intervention. However, as sample sizes increase, this delivery method can become expensive, time and labor intensive, and the data are exposed at many points to the potential for human error [3]. Further, items may be left unanswered by the participant, and follow-up on these items, particularly those that are sensitive in nature, may be uncomfortable and perceived as coercive by participants.

### The Potential Role of Technology in Questionnaire Delivery

Technological advances continue to generate new devices that, when used properly, may increase the efficiency and accuracy of questionnaire data collection. By delivering questionnaires digitally, researchers pay fixed costs initially (eg, development costs, equipment costs), and unlike paper-based questionnaires, these costs increase little with expanding sample sizes [3-5]. Digital questionnaire delivery provides several additional advantages: (1) data are able to be validated in real time, (2) prompts can be provided where necessary for completion of missing or unreasonable items, and (3) the need for a data-entry process is alleviated, removing another potential source of error.

The advantages of digital questionnaires over paper-based questionnaires are of little value if the intended audience reacts negatively to their use. Indeed, the rapid evolution of technology, particularly with regard to input styles and techniques, can make it difficult for some individuals to adopt new devices [6]. For example, for most of their adult lives, older adults were not exposed to computer technology. Most older adults were never employed in a position that required computer proficiency, nor trained in such skills [7]. For these individuals, Internet-based questionnaires delivered via personal computer (PC), which require the use of a mouse, keyboard, and Internet browser come with a steep learning curve [8-12]. Fortunately, advancements in tablet computer technology in recent years have produced devices that lessen these barriers. Tablet computers are equipped with sensitive touchscreens, resulting in a device that is more intuitive and interactive (ie, does not require an input device) [8,12,13]. Further, these devices typically do away with complicated menus and task bars, and this reduction in visual clutter is an important consideration when designing interfaces for older adults [9]. Such simplicity has likely driven increased rates of tablet computer adoption among older adults. Currently, in those 50-64 years of age, 27% now own a tablet computer, as do 13% of those greater than 65 years of age, an increase from 4% and 2% respectively in 2010 [14,15].

Results from the few interventions using tablet devices to positively influence health behavior in older adults have been promising [16-18]. For example, Silveira et al [17] delivered a 12 week exercise intervention to three groups of older adults: Two groups received a tablet-based goal setting and self-monitoring application, and one tablet group received the intervention plus a social networking component. A third completed all study activities with printed materials. Those in the tablet-based groups demonstrated greater adherence and engagement with the program, and those who received the application with a social component were more likely to change their behavior than those who received the print-based materials.

These studies suggest that tablet computers can be used to effectively deliver content to older adults. However, the apparent feasibility of tablet-based questionnaire delivery within this population, and the potential advantages of doing so, do not provide a sufficient basis for the adoption of the technology in the research context. Similar concerns were expressed when Web-based, PC-delivered questionnaires initially increased in popularity. For example, several researchers examined whether mode effects existed for Web-based versus paper-based delivery of questionnaires [19-22]. Denscombe [19] conducted a direct comparison between Web-based and paper-based questionnaires delivered to teenage students, and found little evidence that a mode effect was present.

Because tablet computers are able to offer unique, minimally cluttered interfaces, and because they provide a unique method for interaction, it is important to determine whether these features result in improved or diminished experiences for potential users. It is also important to determine whether these features influence the content of the data collected. The purpose of this pilot study was to compare tablet computer and printed questionnaires as vehicles for the collection of psychosocial data from older adults to determine whether this digital platform would be readily adopted by the sample, and to identify whether mode effects are present. It was hypothesized that a significantly greater proportion of an older adult sample would prefer the tablet-delivered questionnaires, and it was also hypothesized that the collected data would not vary due to mode of delivery.

## Methods

### Design of Questionnaire and Inventory Evaluation via Tablets

The present study implemented a proprietary Web-based software package designed for the Apple iPad 2, with an interface that was customized for older adults (eg, large font sizes; high contrast between text, selected answer, and background; minimization of visual clutter) [9]. A battery composed of three questionnaires that include a wide range of answer types was selected to best capture differences in delivery media. The Barriers Self-Efficacy Scale (BARSE) [23] includes 13 Likert-type items, which ask the participants to rate their perceived ability to exercise at least three times per week in the face of various barriers. The Physical Activity Scale for the Elderly (PASE) [24] evaluates a number of leisure time sedentary and physical activity behaviors, and includes a number of conditional items. Finally, the Pittsburgh Sleep Quality Index (PSQI) [25] assesses quality of sleep and sleep disturbances. The PSQI also contains conditional items, as well as a number of short free-response items. For the purposes of the questionnaire and inventory evaluation via tablets (QuIET) study, these three questionnaires were provided in a counterbalanced order to the participants in both print and digital formats.

**Figure 1:**
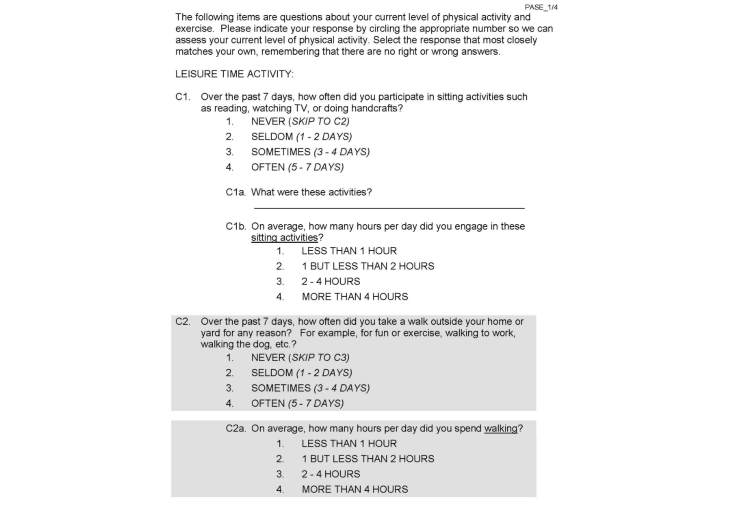
Sample printed questionnaire.

The QuIET study software package was developed as a Web app with the use of hypertext markup language (HTML), cascading style sheets (CSS), JavaScript, and Perl programming languages. The package was designed aesthetically to resemble a pad of paper, and all navigation features were removed to simplify the interface. For short answer questions, participants used the iPad’s digital keyboard to enter responses, and for Likert-type questions, participants were instructed to use a finger or stylus to touch their answer, which highlighted in response. Unlike printed questionnaire completion, for which users are able to gauge progress based on the number of pages completed or remaining, digital questionnaire completion offers no such physical indication of progress through the set. To account for this, a progress bar was included at the top of each page to indicate the portion of the total questionnaire battery completed, and a small motivational prompt indicating percent completion was provided between questionnaires. Though basic aesthetic elements were stylized to enhance clarity, the general layout and content of each questionnaire was the same in the print and digital versions. Figure 1 shows a sample of a printed questionnaire.

**Figure 2:**
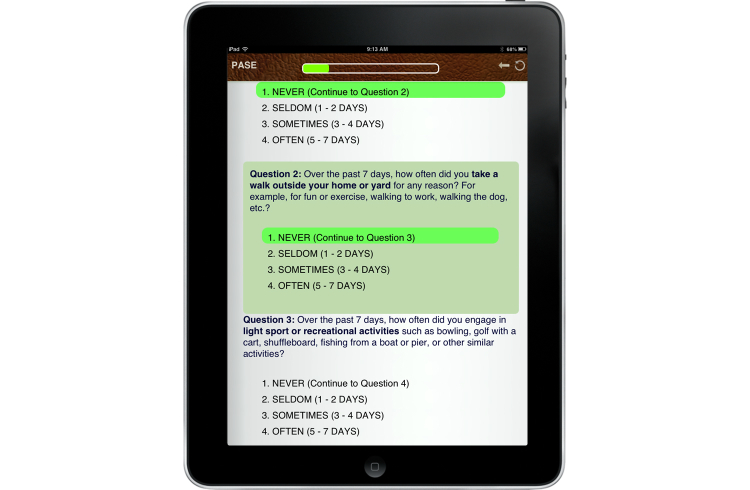
Sample questionnaire without follow-up items.

**Figure 3:**
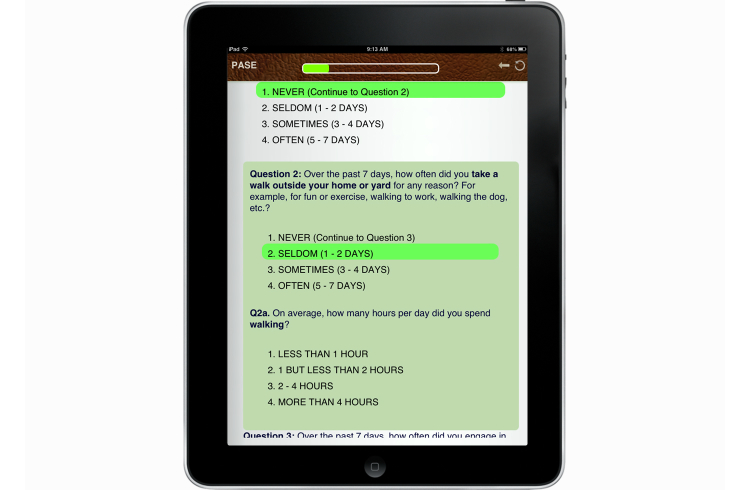
Sample questionnaire with follow-up items.

To further enhance clarity, each questionnaire displayed only appropriate information when presenting conditional items. For example, when asking a question about the frequency with which participants engaged in walking behaviors, possible choices included: (1) Never (Skip to next question), (2) Seldom (1-2 Days), (3) Sometimes (3-4 days), or (4) Often (5-7 days). While the question remained unanswered or if the participant chose “Never”, the questionnaire only displayed the next question (Figure 2 shows a screenshot of this display). Should one of the remaining three options be selected, a follow-up question was displayed asking about the number of hours per day spent doing the activity (Figure 3 shows a screenshot of this display). This was intended to reduce confusion and errors associated with incorrectly answering conditional questions (eg, answering follow-up questions when not applicable).

To improve accuracy, user input was validated upon submission of each questionnaire. This validation was accomplished with JavaScript and Perl. In the case that a question was left unanswered, a prompt was given to the participant that alerted them to the specific question missed. They were given the option to answer the question or to skip it if they were unable to provide an answer. If a question was answered and a required follow-up question was skipped (eg, “How many hours did you do this activity?”), the user was alerted to the missed item and was unable to proceed until it had been answered. All numeric free-response items were also checked for plausibility. For instance, for an item inquiring about number of hours of sleep per night, if a participant entered a number greater than 24, they were prompted to revisit the question and edit their answer before being allowed to continue. After all data were validated, the participant was allowed to proceed to the next questionnaire.

### Recruitment, Screening, and Randomization

Due to the nature of the primary research question (ie, will a significantly greater proportion of a sample of older adults prefer digitally delivered questionnaires), an estimated minimum of 38 participants was needed to detect whether 75% of participants preferring the digital questionnaire was significantly different from 50% preferring each method. This estimate was calculated at a 5% level of significance, 80% power (two-sided test), and with a dropout rate of 25%.

A number of methods were used to recruit community dwelling older adults. Short recruitment talks were given to local older adult philanthropy groups, life-long learning program participants, and senior exercise group participants. Additionally, emails were sent to individuals in existing study databases that agreed to participate in future research. Eligible individuals were English speaking, free from cognitive impairment as assessed via the Modified Telephone Interview for Cognitive Status [26], and willing to attend a single session at the research center.

Depending on individual preference, participant screening was conducted on the Internet or by telephone. During the initial screening process, demographics as well as information pertaining to current computer and mobile device use habits were collected. This included the type of mobile devices used (ie, smartphone, tablet, e-reader), number of hours the computer and mobile devices were typically used, and the reason for using these devices. After this screening process, participants registered for a one hour appointment at the research center. Upon recruitment closure, participants were randomly assigned to one of two groups: (1) the iPad-first group that received the tablet-based questionnaires prior to receiving the paper-based questionnaire packet, or (2) the paper-first group that received the printed questionnaires first. Due to the small number of questionnaires provided, participants received and completed their second set of questionnaires upon completion of the first.

### Measurement and Evaluation

At the end of the testing session, an evaluation of the process of questionnaire completion was given to each participant. Using a four point scale, participants were asked to rate ease of use, as well as their perceived level of arousal for each delivery type. They were asked to comment on strengths, weaknesses, and features to change for each medium. Finally, they were asked to indicate which method they preferred. All data were compiled and analyzed using SPSS version 21 for Windows [27]. Study protocols were reviewed and approved by a university Institutional Review Board, and all participants signed an informed consent document.

## Results

### Participants

A total of 56 individuals responded to recruitment efforts. There were two individuals that were too young to participate, one was unable to attend a session at the study center, and four qualified to participate, but did not attend their scheduled session. The participants who completed the study (N=49; median age 64; interquartile range [IQR]=57-71) were mostly female (36/49, 74%), caucasian (39/49, 80%), and well educated (21/49, 43% with a graduate degree; see Table 1). Greater than half of the study participants used a mobile device daily (n=25), 94% used a computer daily (n=46), 13 owned a smartphone, and 18 owned a tablet computer or e-reader (see Table 2).

### Preferred Method of Questionnaire Delivery

A nonparametric one-sample binomial test indicated a significantly greater proportion of individuals preferred the tablet-delivered questionnaires to the traditional pen and paper method (*z*=4.96, SE 3.428, *P*<.001). Normally distributed scale scores were compared using paired-sample *t* tests, and Wilcoxon sign-rank tests were used to compare scale scores that were not normally distributed. These tests indicated that measures collected by each method were not significantly different (all *P*≥.273; see Tables 3 and 4).

The association between preferred delivery method and daily mobile device use approached significance (*r*_*s*_=.28, n=47, *P*=*.*06), and the association between preferred delivery method and daily computer use was significant (*r*_*s*_=.42, n=47, **P*<.*05), such that those who preferred paper delivery were less likely to use a mobile device or computer each day. The perceived ease of use of the tablet interface, as well as reported anxiety while completing the digital questionnaires, were also significantly correlated with preferences (*r*_*s*_=.665, n=47, **P*<.*001 and *r*_*s*_=.552, n=47, *P*<.001, respectively), indicating that those who preferred the digital delivery method were more likely to find it easier to use and were less anxious while using it. Preferred delivery method was not significantly correlated with the remaining device-use variables (ie, number of hours of daily computer use, type of mobile device owned, hours of mobile device use), perceived ease of use of the paper packet, or reported anxiety felt while completing printed questionnaires.

With regard to strengths of the tablet delivery, participants most frequently noted improved speed of use (n *=* 16; eg, “Was quicker than writing. Didn’t get messy”) and ease of entry (n *=* 8; eg, “Very easy to choose answers”). The most commonly cited weaknesses were related to navigation (n=5; eg, “I had trouble getting used to scrolling”) and formatting (n=5; eg, “Difficulty with time [input] box”).

Regarding paper delivery, commonly noted strengths related to readability (n=14; eg, “Can see everything at the same time”) and navigation (n=7; eg, “Can skip ahead, look back”). The participants most commonly stated that the paper packet was time consuming or cumbersome (n=8; eg, “Seemed more time consuming and longer”) and was not dynamic (n=6; eg, “Not clear that you could skip questions if you hadn't answered initial question”).

**Table 1:** Sample demographics.

Variable		PF^a^, n	IF^b^, n	χ^2^	Total, N=49	df^c^	*P*
**Sex**				.004	49	1	.947
	Male	7	6				
	Female	19	17				
**Race**				0.3	42	1	.607
	Caucasian	19	20				
	African American	1	2				
	Not reported	6	1				
**Ethnicity**				0.9	42	1	.335
	Hispanic or Latino	0	1				
	Not Hispanic or Latino	20	21				
	Not reported	6	1				
**Education**				2.4	42	1	.448
	<High school graduate	1	0				
	High school graduate	0	1				
	College or vocational school degree	8	11				
	Graduate level degree	11	10				
	Not reported	6	1				
**Income**				3.9	42	6	.701
	< US $40,000 per year	4	4				
	> US $40,000 per year	9	14				
	Prefer not to answer	7	4				
	Not reported	6	1				

^a^PF=Paper-first group

^b^IF=iPad-first group

^c^df=degrees of freedom

**Table 2:** Sample device use.

Variable		PF^a^, n	IF^b^, n	χ^2^	Total, N=49	df^c^	*P*
**Use computer daily**				0.2	49	1	.626
	Yes	24	22				
	No	2	1				
**Use mobile device daily**				3.5	49	1	.062
	Yes	10	15				
	No	16	8				
**Use smartphone**				0.3	49	1	.560
	Yes	6	7				
	No	20	16				
**Use tablet or e-reader**				0.8	49	1	.357
	Yes	8	10				
	No	18	13				

^a^PF=Paper-first group

^b^IF=iPad-first group

^c^df=degrees of freedom

**Table 3:** Scale scores for digital and print questionnaires.

Scale score	Digital, mean (SD)	Print, mean (SD)	N	95% CI^a^ for mean difference	*t* ^c^	df^b^	*P*
PASE total score	139.43 **(**68.50)	140.50 (68.33)	49	-7.15, 5.00	-0.356	48	.724
BARSE total score	58.95 **(**25.04)	59.18 **(**24.56)	49	-1.66, 1.19	-0.332	48	.741

^a^CI=confidence interval

^b^df=degrees of freedom

^c^Student’s *t* test

**Table 4:** Scale scores for digital and print questionnaires.

Scale score	Digital, median (IQR)	Print, median (IQR)	N	*z* ^a^	*P*
PSQI global score	6.00(3.00-8.00)	5.00**(**2.00-8.00)	49	1.096	.273

^a^Wilcoxon sign-rank test

## Discussion

### Principal Findings

This study provides preliminary evidence in support of the use of contemporary tablet computer devices for collecting psychosocial questionnaire data. The results indicated that a significant majority of older adult participants preferred tablet delivery of the questionnaires. Participants did not respond differently to questionnaire items based on the method of delivery, a finding that is in line with previous research [4]. Surprisingly, despite relatively few individuals owning tablet computers or e-readers, study participants frequently indicated that they felt the tablet-based questionnaire battery was faster and easier to use than the paper packet.

Importantly, findings from this pilot study indicate that older adults may respond positively to and indeed prefer completing digital questionnaires on tablet devices equipped with software like the package designed for this study. Such dynamic and interactive user environments might benefit participants tasked with completing lengthy questionnaire batteries. For example, hiding unneeded follow-up questions on conditional items resulted in a questionnaire that appeared shorter, a finding reflected in user testimonials. Customized and informative prompts can provide motivation and information between questionnaires [5], and the removal of input devices (eg, keyboard and mouse) creates an environment where entry is more natural and intuitive, even for those less familiar with computer technology [8]. Finally, because data are validated as they are entered, participants are able to explicitly and privately state whether they intended to leave a question unanswered, allowing them to avoid being approached to answer potentially sensitive items.

Researchers also benefit from such computerized methods of data collection. Digital data collection removes the need for research staff to manually enter data, and real time validation ensures that collected data are accurate. Although Web-based data collection conducted via PC can take advantage of some of these same features, the interactive nature of touchscreen devices and the ability to create a simple, clutter-free interface allow researchers to deliver a user experience that is likely more comfortable for many older adults. Finally, because Web apps are cross-platform compatible, and because older adults are increasingly purchasing tablet computers, the ability to deliver questionnaires in a digital format may make it easier for researchers to collect data from broader and more diverse populations.

### Strengths

We believe that this study possesses several strengths. It provides preliminary evidence that with the use of a population-specific interface, older adults may find tablet computer-delivered questionnaires to be acceptable, and perhaps preferable to traditional printed methods. Additionally, this study successfully implemented a tablet-based Web app to collect psychosocial questionnaire data. In the context of questionnaire delivery, we believe that the use of a Web app provides several important advantages to the researcher. First, it can be readily designed to be cross-platform compatible, allowing owners of a variety of tablet computer platforms to access study materials. Further, because Web apps do not require that users install software on their device, researchers need only provide study participants a hyperlink to access questionnaires. This may allow researchers to recruit from a broader geographic area without requiring that participants visit the research center.

This study also provides early evidence to suggest that data collected via tablet computer do not statistically differ from those collected with printed questionnaires. Due to the unique characteristics of the platform, this finding is an important first step in establishing the utility of the device in the research context.

### Limitations and Future Directions

It is important, however, to recognize the limitations of this study. First, the sample was primarily female (36/49, 74%). There are, however, proportionately more women than men in the older adult population, and this gender makeup is similar to that seen in many health-related randomized controlled trials [28,29]. The sample recruited for this study also tended to be well educated, and a large proportion (46/49, 94%) used a computer on a daily basis. Accordingly, these individuals may be relatively tech-savvy in comparison with the general population of older adults in the United States, of which roughly half report using the Internet [30]. This may limit the generalizability of the findings, as well as our ability to draw definitive conclusions. Further research targeting lower income and less educated individuals is warranted, as these groups are the least likely to use computer or Internet technologies [31,32]. Additionally, relative to those who did not use a mobile device each day, those who used a mobile device daily appear to be more likely to prefer the tablet-based delivery method. It has been suggested that older adults’ self-efficacy for engaging with and learning about new technology develops in response to previous experiences (eg, in the workplace) and to the environment [6]. It may be beneficial to extend this work further by examining how psychosocial factors, such as self-efficacy, as well as physical factors such as visual or memory impairment, may influence these preferences.

Regarding program design, the decision to require individuals to complete any question should not be taken lightly, as individuals may have valid reasons to leave a question unanswered. In the context of the current study, only questions which provided clarification for an initial question (eg, the number of hours spent in an activity) were required in order to avoid such conflicts, while all other items allowed participants to explicitly state their intent to leave the item unanswered. Finally, it is possible that the progress bar and short motivational messages could bias participant responses. Follow-up research may benefit by providing questionnaires with and without these features to examine whether differences are present.

### Conclusions

The findings from this pilot study indicate that psychosocial questionnaires, when designed for older adults and delivered via touchscreen enabled tablet computers, may improve efficiency of data collection and may provide more accurate data for the researcher. Importantly, tablet computer-based questionnaire delivery does not appear to influence the content of the data collected. With the aid of additional research, these digitally delivered questionnaires may prove beneficial to the study of HRQL in older adults.

## Abbreviations

BARSE: Barriers Self-Efficacy Scale
HRQL: health-related quality of life
IQR: interquartile range
PASE: Physical Activity Scale for the Elderly
PC: personal computer
PSQI: Pittsburgh Sleep Quality Index
QuIET: questionnaire and inventory evaluation via tablets
